# Past, current and future Swedish freshwater monitoring from an authority perspective

**DOI:** 10.1007/s13280-014-0557-0

**Published:** 2014-11-15

**Authors:** Bertil Håkansson, Manuela Notter

**Affiliations:** 1 Swedish Agency for Marine and Water Management, Box 11 930, 404 39 Gothenburg, Sweden; 2 Swedish Environmental Protection Agency, 106 48 Stockholm, Sweden

The aims of the Swedish environmental monitoring programs are to describe environmental status, to show if the environmental quality objectives are met, to warn of environmental disturbances and to adapt to new, societally relevant challenges. Long-term, continuous monitoring is required in order to determine if environmental change is caused by human activity or natural variation. The Swedish freshwater monitoring data are unique in the world, both for their long duration (up to 50 years) and broad spatial coverage.

Swedish lakes and streams have been monitored systematically since 1965. Between 1965 and 1977, data were collected under the umbrella of different research projects. The Environmental Protection Agency monitoring programs were established in 1978. Since July 1, 2011, responsibility for the Swedish freshwater monitoring has been divided between the Swedish Agency for Marine and Water Management and the Swedish Environmental Protection Agency.

From a national perspective, the freshwater monitoring programs present the evidence base needed to assess and identify changes in the environmental status of lakes, streams, and groundwater. The long-term freshwater monitoring data provide a basis for national indicators of the environmental quality objectives: *Flourishing lakes* *and streams*, *Good*-*quality groundwater*, *Zero Eutrophication*, *Non*-*toxic environment*, and *Natural acidification only*. The program monitors relatively undisturbed reference waters as well as waters subjected to acidification, eutrophication, and different organic and inorganic contaminants. Both chemical and biological monitoring are used to link the effects of anthropogenic activity to large-scale changes in aquatic ecosystems. Further development of the program is ongoing to include monitoring of biodiversity. The freshwater program produces data with high quality control checks performed at all stages from collection, analysis, and storage. Data from the national program can also be used as references for assessment of the status of lakes, streams, and groundwater in a regional and local perspective and are an important resource for authors, consultants, and researchers in Sweden and elsewhere.

The environmental monitoring is essential for the Swedish environmental work in general. The process of defining and prioritizing environmental policy issues are based on assessments of these data. Environmental monitoring has a strategic role to develop and monitor the Swedish environmental quality objectives. Furthermore, environmental monitoring plays a dynamic role in the development of environmental quality standards and criteria. At the international level, the results of environmental monitoring are basis for reporting and official statistics.

One of the key strengths of the freshwater environmental monitoring program is its adaptive nature. Over the past 50 years, the program has evolved to respond to challenges related to eutrophication, acidification, toxics, and climate change. Further adaptation of the monitoring program is likely to better meet European standards including EU Water Framework Directive requirements. As this *AMBIO* Special Issue goes to press, new monitoring programs have been initiated to better understand the effects of fire on boreal forest waters.

Besides the importance of freshwater monitoring for the environmental work in general, the high quality measurements are of great value for research. It is therefore with pleasure that the Swedish Agency for Marine and Water Management and the Swedish Environmental Protection Agency together with the Swedish University of Agricultural Sciences, SLU helps with funding for this “anniversary issue” where the 50-year monitoring of lakes and streams is paid attention to. Evaluations of data from the Swedish freshwater program have resulted in nine articles in this *AMBIO* issue, which highlights the depth and great scientific value of the environmental monitoring data. Our ambition is to continue to generate similar high quality freshwater monitoring data in the future.

